# Evaluation of a stakeholder advisory board for an adolescent mental health randomized clinical trial

**DOI:** 10.1186/s40900-023-00425-6

**Published:** 2023-03-28

**Authors:** Alicia M. Hoke, Perri Rosen, Francesca Pileggi, Alissa Molinari, Deepa L. Sekhar

**Affiliations:** 1 Department of Pediatrics, Penn State College of Medicine, 90 Hope Drive, A145, Hershey, PA 17033 USA; 2 Garrett Lee Smith Youth Suicide Prevention Grant, Harrisburg, PA USA; 3 Aevidum, Executive Director, Lancaster, PA USA

**Keywords:** Community-engaged research, Stakeholders, Program evaluation, Adolescents and young adults, Engagement evaluation

## Abstract

**Introduction:**

Community engagement in research is widely accepted as best practice, despite gaps in existing frameworks to evaluate its process, context, and impact on research. The Screening in High Schools to Identify, Evaluate, and Lower Depression (SHIELD) study evaluated the use of a school-based major depressive disorder screening tool in the identification of symptoms and treatment initiation among adolescents, and was developed, implemented, and disseminated in partnership with a Stakeholder Advisory Board (SAB). We summarize outcomes of the evaluation strategy applied through our partnership with the SAB and explore gaps in the available engagement evaluation tools for mixed stakeholder populations including youth.

**Methods:**

SHIELD study SAB members (n = 13; adolescents, parents, mental health and primary care providers, and professionals from education and mental health organizations) advised on study design, implementation, and dissemination over a three-year period. Both SAB members and study team members (i.e., clinician researchers, project managers) were invited to quantitatively and qualitatively evaluate stakeholder engagement after each project year. At the conclusion of the study, SAB members and study team members were asked to evaluate the application of engagement principles in overall stakeholder engagement across the study period, using portions of the Research Engagement Survey Tool (REST).

**Results:**

SAB members and study team members responded similarly when evaluating engagement process (i.e., valued on team, voice represented); means ranged from 3.9 to 4.8 out of 5 points across all three project years. Reported engagement within study-specific engagement activities (i.e., meetings, study newsletter) varied from year to year, with some discrepancy between SAB member and study team evaluations. Using REST, SAB members reported the alignment of their experience with key engagement principles the same or higher than study team members. Qualitative feedback at the conclusion of the study generally matched quantitative measures; adolescent SAB members, however, reported disengagement from stakeholder activities that was not accurately or effectively captured in evaluation strategies employed across the study period.

**Conclusions:**

Challenges exist in effectively engaging stakeholders and evaluating their engagement, particularly among heterogenous groups that include youth. Evaluation gaps should be addressed through the development of validated instruments that quantify the process, context, and impact of stakeholder engagement on study outcomes. Consideration should be given to collecting parallel feedback from stakeholders and study team members to fully understand the application and execution of engagement strategy.

## Abstract (summary)

We conducted a study (Screening in High Schools to Identify, Evaluate, and Lower Depression) to understand if an adolescent major depressive disorder screening tool delivered in the school setting aided in the identification of symptoms and treatment. We planned and conducted this study with the guidance of a stakeholders, including adolescents.

At the end of each study year, we sent an evaluation survey to stakeholders to understand their experience, such as how appropriately the study included stakeholders and their perspectives. We also surveyed the team leading the study to understand their perspectives about stakeholder involvement. In general, both stakeholders and the study team reported feeling positive about stakeholder involvement; However, some stakeholders felt less involved as the study moved forward, and for some activities stakeholders and study team did not agree on how much the stakeholders were involved in study activities. Additionally, adolescent stakeholders reported low involvement in the study when completing the final evaluation, which, unfortunately, was not captured in the evaluations conducted in earlier study years.

By evaluating the experiences of stakeholders, along with gathering perspectives of the study team, we were able to understand how well we involved stakeholders. However, additional questions remain unanswered, such as how best to involve adolescents as stakeholders, and how involving stakeholders impacted the results of our study. Evaluation tools to best understand these impacts are needed across the field of community-engaged research to answer these questions for future studies.

## Introduction

Community engagement in research has flourished over the last two decades, creating opportunities to develop, implement, and disseminate research impacting communities and the health of their citizens. National funding agencies such as the Patient-Centered Outcomes Research Institute (PCORI), Centers for Disease Control and Prevention, and National Institutes of Health have laid the groundwork for health researchers to go beyond the traditional models of research “for” the community, migrating toward expectations that research be created “with” the community of interest.

Despite leadership among national funders to prioritize the inclusion of stakeholders in the research process, the research community has lagged in the development of standard, agreed-upon community and patient-engagement frameworks and nomenclature. Key et al. [1] described a continuum of community-engaged research (CEnR), highlighting levels of involvement for community members and academic research partners at each level. This framework serves as a grounding point for CEnR teams to establish the most appropriate strategies to meet the community engagement goals of a study. Majid and Gagliardi [2] also conducted a review of engagement literature to understand varying terms used to describe levels of “meaningful” engagement. Their review also presented strategies expected of health researchers at each level. Several articles have also described key principles of stakeholder engagement in research [3, 4]. For example, Goodman and colleagues [3] described a consensus-building study that included perspectives from national experts in stakeholder engagement and community stakeholders to identify the primary principles that should underpin stakeholder-engagement activities and methodologies. Harrison and colleagues [4] conducted a review of patient engagement in research. This review identified many similarities to the work described by Goodman and colleagues [3], and also highlighted potential other emerging engagement practices in the field. While there is overlap in the suggested strategy and methodology reported in the described literature, there is still discrepancy and a lack of standardization to guide CEnR teams.

The field of CEnR is also in need of a systematic way to evaluate the accepted best practice of engaging stakeholders and patients in research. Esmail et al. [5] conducted a review of literature describing benefits of CEnR, proposing measurable components for process, context, and impact of engagement. More recently, Luger et al. [6] conducted a mapping review of studies that included CEnR evaluation, sorting evaluation strategies into (a) context measures, which evaluate capacity within the community to engage with research (i.e., training, experiences), (b) process measures, focused on group dynamics and general experiences, and (c) outcome, or impact, measures, which can examine both the impact of the engagement on the community partners and the impact of community engagement on the research, itself. Context and process measures were identified most frequently, where outcome measures were less common. These reviews, and others described by Harrison and colleagues [4] also highlight the lack of standardized evaluation tools and the implications these gaps can have on understanding the impact of stakeholder involvement in research.

The Screening in High Schools to Identify, Evaluate, and Lower Depression (SHIELD) study evaluated the use of a school-based major depressive disorder (MDD) screening tool in the identification of MDD symptoms and treatment initiation among adolescents [7]. This randomized clinical trial (RCT) was developed, implemented, and disseminated in partnership with a Stakeholder Advisory Board (SAB). The engagement strategy for this study was governed by PCORI engagement principles. However, gaps in standardized evaluation methodology presented challenges in understanding the impact our SAB had on study activities. This manuscript describes the evaluation strategy (along with challenges) utilized with SAB members in the SHIELD study, summarizes the outcomes of the evaluation strategy, and explores gaps in the available engagement evaluation tools for mixed stakeholder populations including youth.

## Methods

### Participants and setting

As outlined in Hoke et al. [8], the SHIELD SAB included adolescents (2), a parent (1), mental health and primary care providers (2), and professionals from education (3) and mental health organizations (5). The initial SAB was comprised of 11 stakeholders, with 2 additional stakeholders joining for years 2 and 3 (n = 13). The adolescent SAB individuals were members of a high school mental health club. Due to this arrangement, a small, but fluid number of club members fulfilled the adolescent role for the SAB. The SAB met quarterly in a virtual format for 3 years (spring 2019 to fall 2021) and conducted one in-person meeting during the first year. In-person meetings were discontinued with the onset of the COVID-19 pandemic during the second year. SAB members engaged in a variety of activities (Table 1) across the three engagement years. Some activities spanned the project (i.e., SAB meetings, study newsletters), while others were activated and discontinued based on the milestones of the overarching SHIELD study. For example, engagement year 1 occurred during the launch of the RCT, resulting in stakeholder activities that aligned with recruitment and RCT launch (Table 1), where year 3 focused on results dissemination.

**Table 1:** Stakeholder advisory board member activities during SHIELD study 2019–2021

	Year 1	Year 2	Year 3
*Overall SHIELD Study Focus*	*1. RCT recruitment & launch* *2. Planning for qualitative study components*	*1. RCT implementation* *2. Qualitative study implementation* *3. Planning for dissemination*	*1. Data analysis (RCT and Qualitative)* *2. Result dissemination*
Activities *(example actions)*			
SAB meetings *(pre-reading, attend meetings)*	X Quarterly; 3 virtual, 1 in person	X Quarterly, all virtual	X Quarterly, all virtual
Study newsletter for participating schools *(content development; editing/proofing)*	X Two per year	X Two per year	X Two per year
Support recruitment *(leverage school contacts)*	X		
Qualitative study interview guides *(question development; editing)*	X	X	
MDD awareness video *(development of storyline/script; proofing)*		X	X Dissemination
Result dissemination *(lay language guidance)*		X Qualitative	X Qualitative & RCT
Publications and presentations *(co-authorship)*		X	X

*SAB* Stakeholder Advisory Board, *RCT* randomized clinical trial, *MDD* major depressive disorder

Of note, SAB members for this study did not have a direct role in the foundational development of the RCT, however stakeholder feedback was solicited in its development. This is further described in Hoke et al. [8].

### Procedures and instrumentation

SAB members were invited to evaluate their level of engagement at the conclusion of each program year. Surveys were distributed electronically. The annual SAB member evaluation, influenced by similar surveys utilized in Kraschnewski et al.’s [9] studies, included core process evaluation questions asked across each program year regarding (a) SAB member experience (i.e., ability to contribute ideas, meetings were valuable use of time) and (b) perceived impact of their contributions on the research (i.e., voice represented, ability to leverage expertise, valued on the team). Survey items utilized a 5-point Likert scale with increasing numbers indicating more favorable response. In years 2 and 3, SAB members also reflected on their level of engagement (context evaluation) in stakeholder activities (outlined in Table 1; i.e., study newsletter, presentations and publications), using a 4-point Likert scale where increasing numbers indicate higher levels of engagement.

In addition to evaluating SAB member perspectives, study team members (i.e., clinician researchers, project managers) annually evaluated their perceptions of stakeholder engagement. Study team members reflected on opportunities for SAB members to contribute, and the presence of the stakeholder voice in study progress (process evaluation; 5-point scale), along with SAB member engagement in stakeholder activities (outlined in Table 1).

Each annual survey for SAB members and study team members also included open text fields for more specific feedback. All surveys were completed anonymously.

At the conclusion of the study, in addition to the annual evaluation elements previously described, SAB members and study team members were asked to evaluate overall stakeholder engagement (process evaluation) across the 3-year engagement period using portions of the Research Engagement Survey Tool (REST), developed by the Goodman Lab. [3, 10] REST was designed to evaluate application of PCORI’s engagement principles in stakeholder engagement activities-both how well the engagement principles are executed (quality) and how often (quantity) they are exhibited. Segments of the REST tool were selected for inclusion based on the engagement principals applied through engagement activities with our study. We selected the following four principles, and thereby the associated questions, for inclusion: (a) Partner input is vital, (b) Foster co-learning, capacity building, and co-benefit for all partners, (c) Build on strengths and resources within the community or patient population, and (d) Involve all partners in the dissemination process. Questions utilized 5-point scales where higher numbers in each scale represented greater quality or quantity, as applicable. The SAB member evaluation also included open-ended questions to collect reflections on their entire experience and provide recommendations for future engagement. All surveys were completed anonymously.

Data was managed in REDCap (Research Electronic Data Capture) survey. REDCap is a secure, web‐based platform used for data collection purposes by researchers, hosted at Penn State Health Milton S. Hershey Medical Center and Penn State College of Medicine [11, 12].

### Data analysis

All data were summarized (means and standard deviations) using Microsoft Excel. Each data point was averaged within groups (i.e., SAB, Study team) for each annual evaluation to observe change over time. REST questions were analyzed according to guidance provided by the tool developers [13]. Questions in each section of the REST tool utilized (i.e., engagement principles 2, 4, 5, and 7) were averaged across respondents to develop both a quality and quantity score for each engagement principle measured. Informal comparisons between responses from study team versus SAB member respondents were generated.

## Results

A total of 6/11, 9/13, 8/13 SAB members and 8/11, 10/11, 8/11 study team responses were received in response to the annual evaluation in years 1, 2 and 3, respectively.

### Annual SAB member and study team member process evaluation

**Fig. 1:**
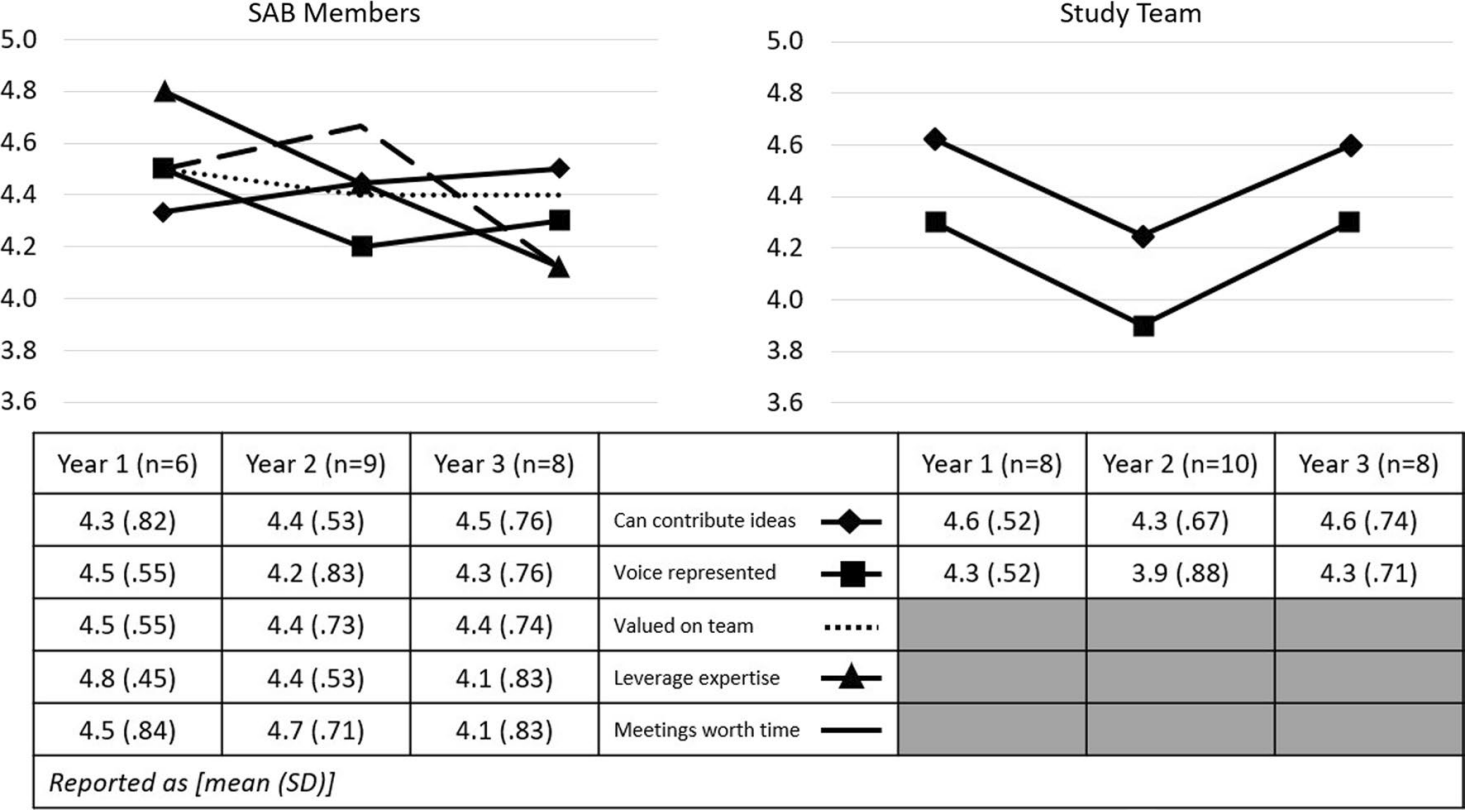
Results from annual engagement process evaluation, as self-evaluated by SAB members and assessed by study team

Overall, SAB members responded favorably about their experience and perceived impact, with a mean score of 4.1 points or higher for each annual process evaluation item (Fig. 1). Study team members responded similarly in their assessment of stakeholder opportunities to contribute ideas and the representation of the stakeholder voice in study progress, reporting a mean of 3.9 points or higher for each process evaluation item (Fig. 1). The inclusion of the stakeholder voice was reported lowest in year two by both SAB members and study team members. The study team evaluation also reflected fewer opportunities to engage SAB members in year two, as compared to years one and three. In addition, SAB members reported an annual decline in the ability of the study team to leverage their expertise, though they perceived an increasing ability to contribute their ideas across the three study years.

### Annual SAB member and study team member context evaluation

**Fig. 2:**
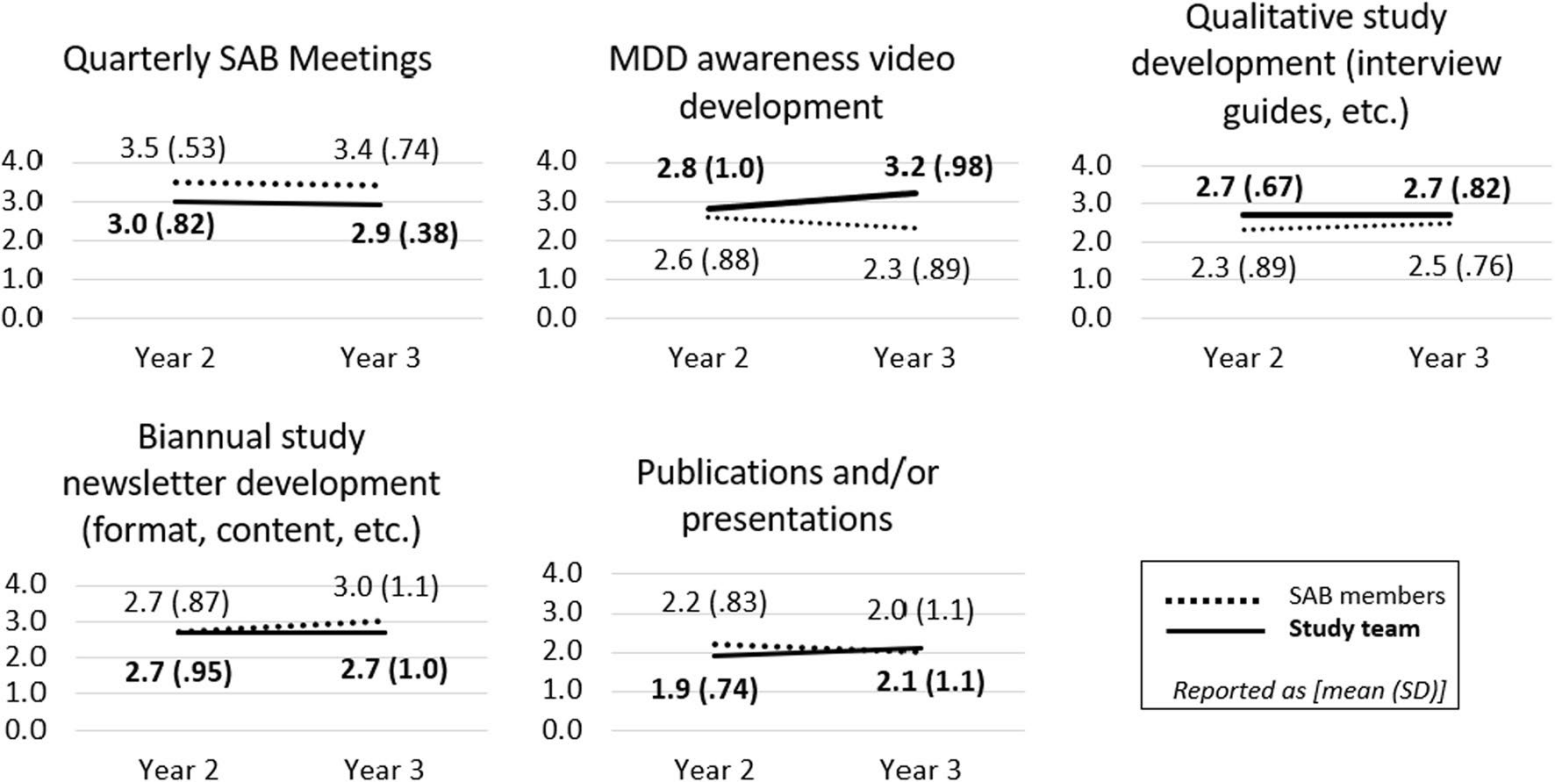
Results from annual assessment of engagement in stakeholder activities, as self-evaluated by SAB members and assessed by study team

SAB members and study team members responded similarly when evaluating levels of stakeholder engagement in stakeholder activities in years 2 and 3, including quarterly SAB meetings, development of a MDD awareness video, development of qualitative study elements (i.e., interview guides), development of the biannual study newsletter (i.e., contributions to format and content), and the development of publications and/or presentations related to the study (Fig. 2). SAB members and study team members reported stability or minor deviations in engagement from year two to three. The most notable discrepancy was engagement with the MDD awareness video, for which SAB members reported a decrease in engagement from year 2 to 3 and study team members reported a perceived increase in stakeholder engagement. Both groups perceived highest levels of stakeholder engagement with quarterly SAB meetings, compared to other activities. Though, SAB members reported slightly higher levels of engagement with quarterly SAB meetings in both years 2 and 3, when compared to study team members. Meeting attendance remained stable across each year (data not shown).

### Overall SAB member and study team member process evaluation-REST tool

**Fig. 3:**
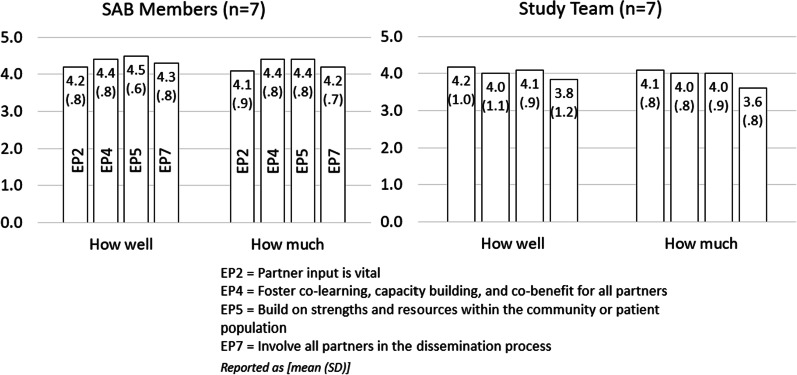
Overall engagement evaluation results, as reported by SAB members and study team members using the Research Engagement Survey Tool (REST); EP-Engagement principle

Using REST, study team members (n = 7) and SAB members (n = 7) evaluated overall engagement strategies across the three years and their alignment with specific PCORI engagement principles (Fig. 3). SAB members evaluated both quality (how well) and quantity (how much) of the principles the same or higher than study team members. SAB members and study team members responded most similarly in their experience of engagement principle 2, “partner input is vital.” For engagement principle 4, SAB members rated the quality of engagement efforts that “Foster co-learning, capacity building, and co-benefits for all partners” higher (4.4) than study team members (4.0). The largest discrepancy between groups was noted in quality and quantity of engagement principle 7, “Involve all partners in the dissemination process”, where SAB members evaluated both quality (4.3) and quantity (4.2) higher than the study team, who averaged quality and quantity scores at 3.8 and 3.6, respectively.

### Qualitative feedback from final evaluation

SAB members were asked to reflect on whether they met their personal goals for serving on the SAB. The majority responded favorably, one member sharing that they were able to “assist with meaningful work that will benefit schools and students” another stating they “learned a lot about research and the rolling out of a research study of this magnitude.” Of those who responded (n = 7), all indicated they would accept an opportunity to serve as a stakeholder on another project. However, feedback from participating students and supporting student advisors indicated that the experience may not have been equally as rewarding or beneficial. One student (as identified in the open text of the anonymous survey) reflected on meeting their goals as “I don’t know. I understood almost none of what anyone was saying, ever.” We also received feedback suggesting the “students felt rather clueless at many meetings…it’s important they feel engaged from the start.” The feedback was surprising, as these concerns were not raised in any previous evaluation timepoints and, therefore were unfortunately unable to be addressed in a timely manner.

## Discussion

Engagement evaluation strategies for the SHIELD study spanned across both context and process domains. The summarized quantitative evaluation data suggest a high degree of stakeholder engagement in designated engagement activities, though challenges emerged in engaging all members of our heterogenous SAB equally. The inclusion of study team member perspectives on stakeholder engagement, in addition to the self-evaluation of SAB members, both corroborates the results, and introduces nuances to understand success and impact of stakeholder engagement that may be missed by a single perspective.

SAB members self-reported engagement with quarterly meetings higher than any other engagement activity. This is understandable considering it was the most accessible way for SAB members to be involved in the study. Engagement in the development of the biannual study newsletter remained high across both years 2 and 3. This is likely because two newsletters were developed each year and we solicited support directly from individual SAB members to serve in both writing and reviewing roles, rotating our requests across SAB members for each edition. Activities that utilized a “request for volunteers” strategy (i.e., qualitative study development) garnered lower rates of reported participation, and thereby, reported engagement. We also recognize that activities that are more typically aligned with academic activities (i.e., publications/presentations) were of less interest to our SAB member group, unless the stakeholder had a personal or professional motivation for partnering on the activity. These observations suggests a need for alternate approaches to describing the activities and additional training may be needed to reduce barriers to participation.

One area for overall engagement improvement was sustainability of engagement over time. Our ability to leverage stakeholder expertise throughout the study period and ensure meetings were worth the time of our stakeholders waned as the study approached completion, despite the fact that meeting participation remained stable and SAB members felt an increasing ability to contribute ideas. This may be a result of stakeholder activities changing across the study period (Table 1) to align with progress of the SHIELD study. As the study moved from development and launch, into implementation, and then toward data analysis and dissemination, the focus of quarterly meetings shifted toward delivering updates and involvement of individual SAB members in some specific engagement elements (i.e., publications), rather than collaboratively developing study products. This is not unique to our project, as others reported similar challenges in sustaining high levels of engagement throughout the course of a study [14–16].

Another interesting finding was the discrepancy between SAB members and study team members in their perceptions of how well and how much the study exhibited alignment with engagement principal 7, which describes engagement of all partners in the dissemination process (Fig. 3). SAB members perceived more and better alignment, suggesting an imbalance in the expectations of the study team and the SAB members, and the need to collaboratively develop expectations at the outset, and revisit them throughout the length of the partnership. While our study’s engagement approach did utilize strategies recommended by previous stakeholder-engaged studies, such as establishing shared expectations and sustaining engagement through frequent study meetings, utilization of study newsletters, and ongoing training and education opportunities [9], it is clear that not all members of our heterogenous SAB were equitably engaged. Our experience suggests that stakeholders may benefit from clearer understanding of the parameters and expectations for each activity, and how the activity ties to the study, along with ongoing and repeated grounding discussions about the status and purpose of the study activities. Additional exploration is warranted to understand the application of these, and other, engagement strategies in varying stakeholder engagement structures and group compositions.

Engaging a heterogenous group of stakeholders is encouraged [17, 18] to ensure the research question is relevant, project implementation is feasible, and dissemination is robust and well received by the impacted community. This is especially true with health research involving youth populations, as literature describes the critical role youth engagement plays in adoption of/participation in health research and outcomes [14, 15, 19]. However, for our adolescent members, end of program evaluation elicited feedback about less than ideal experiences, though these concerns were not well captured through annual evaluation metrics. We hypothesize several explanations for limitations in adolescent engagement. First, quarterly stakeholder meetings were only conducted virtually after year one. While second nature now, the concept of engaging virtually was not the norm when introduced as the only form of stakeholder meeting, necessitated by the COVID-19 pandemic. Thus, adolescents could have joined the meetings but not be fully engaged due to a lack of understanding and/or feeling reluctant to speak up and seek clarification due to the heterogenous backgrounds of participating stakeholders. Second, communication with the adolescents between meetings was indirect as a result of needing to work through a club advisor. This indirect engagement may have impacted the connection or feeling of engagement of adolescent members. Lastly, there was inconsistency among which of the adolescent SAB members attended the quarterly meetings. Although the engagement strategy was designed this way to increase the presence of the adolescent voice in stakeholder activities (i.e., avoiding consistent schedule conflicts with more than one adolescent stakeholder available), this model potentially introduced challenges among adolescent SAB members to fully understand the historical SAB meeting information and stay connected. As such, adolescent SAB members may have lower confidence to engage. Future opportunities to engage adolescents as stakeholders may benefit from a youth leadership role to improve communication with the study team and improve equity among SAB members. The challenges we experienced in including adolescent stakeholders have been described by others, along with possible solutions for best engaging youth [14, 19–21]. Special consideration should be given to the ethical inclusion of youth, particularly in heterogenous stakeholder board scenarios [22].

Evaluation of stakeholder experience in heterogenous stakeholder populations presented unanticipated challenges and opportunities for improvement in the field. Namely, the needs of all stakeholders in a heterogenous stakeholder group may not be synonymous, resulting in the need for diverse engagement evaluation strategies that take into consideration, among other things, varying ages and education. Much of the evaluation literature [23, 24] focuses on utilizing qualitative strategies, which limits comparison within projects and generalizability across projects, but provides a rich understanding of individual stakeholder experiences. Martinez et al. [25] describe a stakeholder-centric instrumentation process where evaluation tools are customized to each project and accompanying stakeholder body. One challenge with the development of project-specific tools is the generalizability of stakeholder engagement, experience, and impact across the field. However, strategies Martinez and colleagues proposed may be helpful in the development of standardized tools that are representative of the interests and values of specific stakeholder groups, such as youth.

The results of our study also present an opportunity to consider standardized strategies and best practices in evaluating engagement and its impact by both the stakeholder group and members of the study team. Development of validated quantitative evaluation tools designed for stakeholder use should have parallel tools to measure study team perspectives on the same topics, thus creating a better understanding of context, process and impact evaluation data. A first step in this process is the development of accepted engagement terminology across study teams and research stakeholders. Key and colleagues, along with Majid and Gagliardi describe formative work in this space. Sanders Thomson et al. [26] describes, more specifically, discrepancies in the way academicians and community members understand and interpret language used in engaged research. For example, the research term “stakeholders” and it’s community member alternate definition, “people with relevant lived experience.” These gaps must be bridged before standardized, inclusive, and meaningful evaluation can occur.

Our experience in engaging stakeholders in the development, implementation, and dissemination of a randomized clinical trial with direct community impact exposed opportunities for improvement in evaluating the process and context of engagement with diverse stakeholder partners, in addition to the value of collecting parallel feedback from study team members. Learnings from our experience should also be considered as the field addresses another evaluation gap-the availability of validated instruments that quantify the impact of stakeholder engagement on study outcomes [23, 27]. Encouragingly, there is movement to bridge this gap. PCORI convened a workshop in 2016 to consider strategies for envisioning impact of stakeholder engagement and its evaluation [28], and Maurer [27] and colleagues conducted a qualitative study with researchers and stakeholders involved in 58 studies funded by PCORI to understand stakeholder engagement impacts on phases of the research process. Most recently, PCORI released a Science of Engagement request for proposals [29] “seeking to fund studies that build an evidence base on engagement in research, including measures to capture structure/context, process, and outcomes of engagement in research.” We look forward to the evaluation opportunities created through this funding mechanism.

### Limitations

Evaluation data presented in this manuscript were self-reported by study team members and SAB members engaged in our study. We acknowledge limitations presented by our sample size and response rates, along with challenges presented by a lack of demographic information about our stakeholders. Additionally, due to the heterogenous nature of our SAB, and the anonymous format of our evaluations, we cannot confirm if the same SAB members participated in evaluation from year to year. We acknowledge that the evaluation data described represents stakeholder perspectives engaged with only one study, and may not be generalizable to other community-engaged research. Within our engagement evaluation strategy, we elected to utilize segments of an existing, albeit imperfectly aligned, evaluation tool, rather than engage our SAB members in the development of new, study-specific tools. Future efforts in engagement evaluation should prioritize the involvement of stakeholder populations in development and testing of instrumentation. These limitations further support the need to collectively move toward accepted terminology and standardized evaluation strategies to improve generalizability in engagement evaluation.

## Conclusions

Engaging community members with varied perspectives and lived experience is an increasingly accepted research practice, however the mechanisms for effectively and consistently evaluating the process, context, and outcomes of those engagement strategies lags behind the practice. Challenges still exist in effectively engaging stakeholders, particularly heterogenous groups that include youth. Further exploration is needed to develop evaluation strategies that include broad (i.e., both qualitative and quantitative) understanding of engagement within a study, in addition to standardized metrics that can be used to understand the impact of engagement across community-engaged research.

## Data Availability

The datasets used and/or analyzed during the current study are available from the corresponding author on reasonable request.

## Abbreviations

CEnR: Community-engaged research
EP: Engagement principle
MDD: Major Depressive Disorder
PCORI: Patient-Centered Outcomes Research Institute
RCT: Randomized Clinical Trial
REDCap: Research Electronic Data Capture
REST: Research Engagement Survey Tool
SAB: Stakeholder Advisory Board
SHIELD: Screening in High Schools to Identify, Evaluate, and Lower Depression
